# Lüftungskonzepte in Schulen zur Prävention einer Übertragung hochinfektiöser Viren (SARS-CoV‑2) über Aerosole in der Raumluft

**DOI:** 10.1007/s00103-021-03452-4

**Published:** 2021-11-05

**Authors:** Wolfram Birmili, Hans-Christoph Selinka, Heinz-Jörn Moriske, Anja Daniels, Wolfgang Straff

**Affiliations:** 1 Umweltbundesamt, Abteilung II 1 „Umwelthygiene“, Corrensplatz 1, 14195 Berlin, Deutschland; 2 Umweltbundesamt, Beratungsstelle Umwelthygiene II BU, Wörlitzer Platz 1, 06844 Dessau, Deutschland

**Keywords:** SARS-CoV‑2, Infektion, Aerosol, Lüftung, Luftreinigung Schulen Innenraum, SARS-CoV‑2, Infection, Aerosol, Ventilation, Air purification schools indoor

## Abstract

Aller Kenntnis nach spielen die mit der Atmung ausgeschiedenen Aerosolpartikel eine wichtige Rolle bei der Verbreitung des 2019 erstmalig aufgetretenen Coronavirus SARS-CoV‑2, insbesondere im Rahmen menschlicher Zusammenkünfte in Innenräumen. Diese Arbeit fasst die für den Schulbetrieb relevanten Sachverhalte und Maßnahmen zur Verminderung von Infektionen über den Aerosolpfad zusammen. Eine wichtige Maßnahme ist die Verstärkung der Raumlüftung, d. h. der Austausch möglicherweise kontaminierter Innenraumluft mit Außenluft. Neben der Verminderung der Konzentration infektiöser Aerosole ist Lüftung unabdingbar zur Abfuhr des in Klassenräumen erzeugten Kohlendioxids, der Luftfeuchte und anderer chemischer Stoffe in der Innenraumluft. Unabhängig von Lüftung erweist sich das Tragen von Mund-Nasen-Masken (medizinische Masken bzw. filtrierende Halbmasken) als wirksame Maßnahme. Eine für virushaltige Partikel wirksame Luftreinigung durch feste bzw. mobile Anlagen kann die genannten Maßnahmen unterstützen bzw. in Fällen aushelfen, wenn Räume trotz schlechter Lüftungsmöglichkeit genutzt werden müssen. Der Artikel gibt den Stand des Wissens im Oktober 2021 über verschiedene technische Schutzmaßnahmen wieder, die sich seit Beginn der Pandemie als sinnvoll erwiesen haben, wobei der Fokus auf der Reduzierung von indirekten Infektionen liegt. Neu hinzukommende Varianten von SARS-CoV‑2, der Fortschritt der Impfkampagne bei Kindern und Jugendlichen sowie die Zunahme der allgemeinen Immunität werden möglicherweise eine Neubewertung der Maßnahmen erfordern. Neben kurzfristigen und schnell wirksamen Maßnahmen zum Infektionsschutz erscheint es auch geboten, die in Deutschland existierenden Defizite bei Raum- und Gebäudelüftung an Schulen durch eine langfristige Strategie zu beheben. Im Sinne einer dauerhaften Verbesserung der Innenraumluft und der Prävention gegen künftige luftübertragene Infektionskrankheiten erscheint die zunehmende Ausstattung von Schulen mit fest installierten Lüftungsanlagen bzw. raumlufttechnischen Anlagen – mit Option auf Wärme- und Feuchterückgewinnung – als nachhaltige gesellschaftliche Investition.

## Abstract (en)

Exhaled aerosol particles play an important role in the transmission of SARS-CoV‑2, particularly when many people gather indoors. This article summarises the knowledge on virus transmission in schools and practical measures to reduce aerosol-driven infections. A central preventive measure is to enhance room and building ventilation, i.e. the exchange of possibly contaminated indoor air with ambient air. Besides the concentrations of possibly infectious particles, ventilation reduces carbon dioxide concentrations, humidity and other chemical substances in indoor air as well. Irrespective of ventilation, face masks (surgical or FFP2) represent a vital part of hygiene measures. Fixed or mobile air purifiers can support these measures particularly when rooms providing only poor ventilation must be utilized. The article reflects the state of knowledge in October 2021 of the various techniques that have been shown as useful for the prevention of indirect infections. New variants of SARS-CoV‑2, the progress of the vaccination campaign in children and adolescents, and the increase in general immunity might require a re-evaluation of the prevention strategies described. The COVID-19 pandemic has revealed common deficits in room and building ventilation, not least in schools. Apart from short-term measures for the prevention of airborne infectious diseases, a long-term strategy seems advisable to alleviate the deficits encountered in schools with respect to room and building ventilation. In view of a permanent improvement of indoor air and prevention against airborne infections the fitting of schools with fixed ventilation systems – preferably including heat and moisture recovery – appears to be a sustainable social investment.

## Einleitung

Dieser Artikel stellt die aktuelle Problematik der Übertragung hochinfektiöser Viren (SARS-CoV-2) über Aerosole in der Innenraumluft vor. Verschiedene, für Schulen geeignete infektionsmindernde Maßnahmen, insbesondere bezüglich der Lüftung von Räumlichkeiten, werden beschrieben und verglichen. Der Artikel schließt mit geeigneten Empfehlungen und einem Ausblick für Lüftungsanforderungen in der Zukunft.

### Die Rolle von Schulen in der SARS-CoV-2-Pandemie

Seit der weltweiten Ausbreitung des SARS-CoV‑2 nimmt die Frage nach geeigneten und angemessenen Maßnahmen zu einer wirksamen Eindämmung der Pandemie eine zentrale Rolle ein. Die begrenzte Fähigkeit der Gesundheitsbehörden, gemeldete Infektionsfälle zurückzuverfolgen macht deutlich, dass die Mechanismen und Orte, an denen das Virus bevorzugt übertragen wird, vielfältig und in ihrer jeweiligen Relevanz nicht gut bekannt sind. Insbesondere zu Beginn der Pandemie gab es viele Unsicherheiten und Kontroversen bei der Planung und Priorisierung infektionsmindernder Maßnahmen im gesamtgesellschaftlichen Kontext.

Zahlreichen Hinweisen zufolge spielen menschliche Zusammenkünfte in Innenräumen eine zentrale Rolle bei der Verbreitung von SARS-CoV‑2 [1]. Mechanismen der Infektion sind die Übertragung virushaltiger Aerosolpartikel bzw. Tröpfchen im Nahfeld einer infizierten Person sowie die Anreicherung und Übertragung virushaltiger Aerosolpartikel in Innenräumen [2, 3].

An Bildungseinrichtungen wie Schulen kommen Menschen verschiedener Altersgruppen über viele Stunden auf engem Raum zusammen. Während Infektionen bei Kindern meistens einen milden Verlauf haben [4], erkranken Erwachsene, wie zum Beispiel das Lehrpersonal und auch die Eltern und Großeltern der Kinder, abhängig von Alter und bestehenden Vorerkrankungen mit deutlich schwerwiegenderen Verläufen [5]. Als Präventionsmaßnahme wurde in Deutschland schon bald die verstärkte Lüftung von Innenräumen diskutiert [6]. Ab März 2020 wurde jedoch in vielen Bundesländern begonnen, Schulen zu schließen bzw. den Unterricht online zu verlagern. Mit Beginn der zweiten Infektionswelle im Oktober 2020 wurden Schulschließungen als schnelle und geeignete Maßnahme zur Verringerung der Infektionsfälle betrachtet.

Die Beurteilung von Infektionsrisiken in Innenräumen entwickelte sich mit dem Wissen über die aerogene Verbreitung der Infektion, der Inzidenz der Erkrankung in der Bevölkerung, dem Auftreten von Virusmutationen [7] und zusätzlich mit ersten Erkenntnissen über die Langzeitfolgen von COVID-19-Erkrankungen bei Erwachsenen [8] und Kindern [9]. Zum Zeitpunkt des Schreibens (Oktober 2021) war die Kenntnis über die Entwicklung der Pandemie und ihre Einflussfaktoren noch immer im Fluss und es war kaum abzusehen, wann aufgrund von Impfungen und steigender Immunität in der Bevölkerung jegliche Präventionsmaßnahmen aufgehoben werden können.

### Virushaltige Partikel in der Raumluft

Die respiratorische Aufnahme von Aerosolpartikeln^1^ gilt als Hauptübertragungsweg für SARS-CoV‑2 [1]. Mit der ausgeatmeten Luft verbreitet jeder Mensch auch Aerosolpartikel in seiner unmittelbaren Umgebung. Wie bei nahezu allen Atemwegserkrankungen, die mit typischen Symptomen einhergehen, scheiden infizierte Personen Partikel aus, welche die Krankheitserreger enthalten. Eine Besonderheit von SARS-CoV‑2 besteht darin, dass auch infizierte Menschen ohne Krankheitssymptome über einen Zeitraum von mehreren Tagen virushaltige Partikel ausscheiden können. Dies hat unter anderem dazu geführt, dass sich in der Anfangsphase der Pandemie viele Personen auf diesem Weg infiziert haben.

SARS-CoV-2-Einzelviren haben Durchmesser im Bereich 0,06–0,14 μm [10]. Sie werden in der Regel als Bestandteil größerer wässriger Partikel ausgeatmet, welche sich in Abhängigkeit der Umgebungsbedingungen (relative Luftfeuchte und Temperatur) bezüglich ihres Durchmessers und Wasseranteils ändern können [3, 11]. Im medizinischen Sprachgebrauch werden größere, teilweise gerade noch sichtbare, Aerosolpartikel häufig als „Tröpfchen“ beschrieben und solche kleiner als 5 µm als „Aerosol(partikel)“. Physikalisch handelt es sich bei beiden jedoch um Aerosolpartikel, bei denen vor allem die Partikelgrößenverteilung ausschlaggebend ist für deren Verhalten im Innenraum, die Möglichkeit der Inhalation und die Eindringtiefe in die Atemwege [12]. Anzahl und Durchmesser der ausgeatmeten Partikel hängen stark von der Art der menschlichen Aktivität ab: Bei ruhiger Atmung entstehen vorwiegend kleine Partikel (< 5 µm), beim Sprechen, Rufen, Singen oder unter körperlicher Anstrengung insgesamt vermehrt Partikel und beim Niesen und Husten zusätzlich größere Partikel bis zu einer Größe von 100 µm [13–15]. „Feuchte Aussprache“ erzeugt noch größere, mit dem Auge sichtbare Speicheltropfen. Diese Informationen sind von hoher Bedeutung für die Bewertung der Situation an Schulen, wo Schülerinnen, Schüler und Lehrerpersonal eine große Bandbreite an Aktivitäten verfolgen.

In dicht belegten Innenräumen können virushaltige Aerosolpartikel zum Risiko werden, wenn sie sich bei begrenztem Luftaustausch im Raum anreichern. Während größere Partikel im Bereich von 100 µm innerhalb von Sekunden zu Boden sinken, können Partikel kleiner als 10 µm viele Minuten und Stunden in der Luft verbleiben [12, 16]. Größere Partikel bzw. Tröpfchen kommen daher nur für eine luftgetragene Infektion im Nahbereich einer infizierten Person infrage, wogegen kleinere Partikel, die sich mit der Luftströmung im gesamten Raum verteilen können, für Infektionen sowohl im Nah- wie im Fernfeld sorgen können. In allen Fällen ist davon auszugehen, dass im Nahfeld (< 1,5 m) einer infizierten Person höhere Konzentrationen an möglicherweise infektiösen Partikeln anzutreffen sind. Die genauen Prozesse, die zur Emission, zur Ausbreitung und zur Veränderung der ausgeschiedenen Aerosolpartikel führen, sind von einer Vielzahl unterschiedlicher Faktoren abhängig und bislang im Einzelfall kaum vorherzusehen.

### Infektion über Aerosole bei Kindern

Hinsichtlich der Infektiosität bei Kindern bestehen noch zahlreiche Unklarheiten. Beispielsweise ist nicht bekannt, welche Altersgruppen die höchste Infektiosität aufweisen, wobei angenommen werden kann, dass Kinder weniger infektiös sind als Erwachsene [1]. Problematisch im schulischen Kontext ist, dass die Mehrzahl der Kinder nach bisheriger Studienlage einen asymptomatischen oder milden Krankheitsverlauf zeigt [17], wodurch unerkannte Infektionen wahrscheinlicher werden. Allerdings legen Erkenntnisse nahe, dass Schulkinder deutlich weniger zu Infektionen in Schulräumen beitragen als das Lehrpersonal [18] und dass Infektionen beim Aufenthalt in Schulräumen – bei Einhaltung der AHA + L-Regeln (Abstand einhalten, Hygieneregeln beachten, im Alltag eine Maske tragen und Lüften) – insgesamt selten sind [19].

Grundsätzlich müssen folgende Bedingungen erfüllt sein, damit eine Person über SARS-CoV-2-haltige Aerosolpartikel infiziert werden kann:

1. Die Menge infektiöser SARS-CoV-2-Viren im Aerosol muss groß genug sein, damit es bei Inhalation des Aerosols zu einer Infektion kommen kann. Die in die Umgebungsluft ausgeschiedene Menge an Viren ist von individuellen Faktoren abhängig und schwankt je nach Umweltbedingungen und Infektiosität der infizierten Person um mehrere Größenordnungen.
2. Virushaltige Partikel müssen auf empfindliche Zellen treffen, wie z. B. auf Schleimhautzellen der Atemwege, der Augenbindehaut oder der Lunge.
3. Es muss zu einer Vermehrung des Virus in diesen Zellen kommen. Die hierfür notwendige Menge an Viren (Infektionsdosis) kann derzeit nur grob eingegrenzt werden und ist vermutlich ebenfalls von vielen individuellen Faktoren wie dem Immunstatus abhängig. Im Vergleich zu vielen anderen infektiösen Viren wird die Infektionsdosis von SARS-CoV‑2 als geringer eingeschätzt [20], wobei anfänglich eine Größenordnung von etwa 100 Viruspartikeln angenommen wurde [21]. Die Übertragungswahrscheinlichkeit variiert zusätzlich zwischen den unterschiedlichen Varianten von SARS-CoV‑2. So geht man von einer deutlich höheren Transmissionsrate der sog. Deltavariante (B.1.617.2) im Vergleich zur sog. Alphavariante oder zu den zu Beginn der Pandemie vorherrschenden Varianten aus [22, 23].

Aufgrund derartiger Überlegungen und Erkenntnisse stand seit Beginn der Pandemie in Schulen die Infektionsprophylaxe durch gute Belüftung der Klassenräume und gleichzeitigen Einsatz von Mund-Nasen-Bedeckungen und Halbmasken im Vordergrund. Auch die Reduzierung von Klassenstärken gehörte zu den Infektionsschutzmaßnahmen in Innenräumen. Bestimmte Maßnahmen, denen am Anfang der Pandemie noch hohe Bedeutung zugemessen wurde, wie die Händedesinfektion und die konsequente Einhaltung eines Mindestabstandes von 1,5 m, wurden seit der zweiten Pandemiewelle in Deutschland weniger stringent verfolgt, wenngleich sie ihre Gültigkeit behielten. Ein Hauptaugenmerk an Schulen war spätestens seit Herbst 2020 die Entfernung von virushaltigen Partikeln aus der Raumluft durch Maßnahmen wie Lüftung.

## Infektionsmindernde Maßnahmen

### Grundsätzliche Präventionsmaßnahmen

Eine Infektion durch Einwirkung infektiöser Aerosolpartikel, die sich in der Innenraumluft ausgebreitet haben, gilt als „indirekt vermittelte Infektion“. Im Gegensatz hierzu gilt die Übertragung im Nahfeld von Mensch zu Mensch als „direkt vermittelte Infektion“. In der Praxis ist eine Unterscheidung dieser Mechanismen gerade in Schulräumen zur Ableitung entsprechend wirksamer Infektionsschutzmaßnahmen nicht zielführend, weil beide Infektionsmöglichkeiten nicht sicher voneinander getrennt bewertet werden können [24]. Das Robert Koch-Institut (RKI) betrachtet die Anreicherung von SARS-CoV-2-haltigen Partikeln in schlecht belüfteten Innenräumen als einen wichtigen Pfad für eine indirekte Infektion [1]. Die Zeitdauer, über die ausgeatmete SARS-CoV-2-Viren infektiös bleiben, wird mit einigen Stunden abgeschätzt [25]. Dies wird als ausreichend betrachtet, um während der Dauer von schulischen Veranstaltungen eine Rolle zu spielen. Tierversuche und epidemiologische Beobachtungen deuten darauf hin, dass die Inhalation virushaltiger Partikel gleich welcher Größe Infektionen verursachen kann, selbst wenn der genaue Mechanismus der Inhalation bzw. Deposition auf Schleimhäuten noch nicht quantifiziert werden konnte [26].

Trotz dieser Wissenslücken legen die im Laufe der Pandemie gewonnenen Erkenntnisse nahe, dass die zum Zeitpunkt des zweiten Lockdowns (Winter 2020/2021) ergriffenen Präventionsmaßnahmen Infektionen mit SARS-CoV‑2 wirksam verhindert haben. Zu diesen Maßnahmen gehörten die Vermeidung körperlicher Kontakte, der Einsatz gut sitzender Masken, ausgiebige Raumlüftung selbst bei winterlichen Außentemperaturen und die Vermeidung von Menschenansammlungen in Innenräumen. Als weitere Präventionsmaßnahmen galten weiterhin die Desinfektion der Hände und der Kontaktoberflächen im Rahmen der allgemeinen Hygienemaßnahmen [26].

Aus den bis dato bekannten Eigenschaften von SARS-CoV‑2 und dem Verhalten luftgetragener Partikel in Innenräumen lässt sich ableiten, dass das Infektionsrisiko über Aerosole in einem Innenraum umso niedriger ist,

- je weniger Personen sich im Raum aufhalten,^2^
- je kürzer die Personen sich im Raum aufhalten,^3^
- je weniger aerosolbildende Aktivitäten wie Sprechen, Rufen, Singen etc. stattfinden,^4^
- je mehr gut sitzende, dicht abschließende und gut filtrierende Masken getragen werden,^5^
- je größer das Volumen des genutzten Innenraums ist,^6^
- je höher die Luftwechselrate im Raum ist (Austausch gegen nicht virushaltige Luft).

Diese Maßnahmen sind immer im Kontext der grundlegenden Präventionsmaßnahmen wie der AHA + A + L-Regeln (Tab. 1) zu sehen, welche die direkte Infektion wirksam unterdrücken.

**Table 1:** 

Maßnahme	Wirkung	Zu beachten	Quellen
Abstand halten (*A*HA + A + L)	Vermindert die direkte Infektion durch virushaltige Partikel (innerhalb 1,5 m Abstand)	Aktivitäten wie lautes Sprechen, Singen und Schreien können größere Sicherheitsabstände als 1,5 m erfordern	[1]
Händedesinfektion (A*H*A + A + L)	Vermindert Kontakt- und Schmierinfektionen	Geringer Bezug zur Raumluft	[1, 27]
Tragen von Schutzmasken (AH*A* + A + L)	Medizinische Masken (OP-Masken) behindern den direkten Ausstoß von Partikeln in den Raum	Medizinische Masken (OP-Masken) wirken primär im Nahfeld als Fremdschutz	[1, 28, 29]
	Eng anliegende Halbmasken wie FFP2 haben auch eine Filterwirkung gegen Aerosole im Raum	Eng anliegende Halbmasken wie FFP2 wirken als Fremd- und Selbstschutz	
Corona-Warn-App (AHA + *A* + L)	Digitale Nachverfolgung von Infektionsketten	In Innenräumen wie öffentlichen Verkehrsmitteln sinnvoll, da hier der Abstand von 1,5 m häufig nicht eingehalten werden kann Smartphone mit aktiviertem Bluetooth erforderlich	[1] sowie https://www.coronawarn.app
Lüften (AHA + A + *L*)	Verringert die Anreicherung virushaltiger Partikel in der Raumluft	Reduzierung des Risikos einer indirekten Infektion durch Aerosole in Innenräumen. Besonders wichtig in Räumen mit dichter Personenbelegung	[30]

### Lüftungsmaßnahmen

Ein Ersatz der verbrauchten Raumluft gegen Außenluft ist eine bekannte und wirksame Methode, um ausgeatmete Aerosolpartikel abzuführen und somit einer luftgetragenen Infektion entgegenzuwirken. Für den Infektionsschutz hat es keine grundsätzliche Bedeutung, ob derselbe Luftaustausch etwa durch freie Lüftung (Fenster) oder über raumlufttechnische (RLT-)Anlagen^7^ bewerkstelligt wird. Während RLT-Anlagen gleichmäßige Lüftungsbedingungen ermöglichen, wird die Wirksamkeit freier Lüftung von äußeren Bedingungen wie dem Temperaturunterschied zur Außenluft sowie von Windbewegungen beeinflusst. Ein entscheidender Parameter ist die sogenannte Lüftungseffizienz, die man als Reinigungsleistung der Innenraumluft bezogen auf die zugeführte unkontaminierte Luftmenge beschreiben kann. Je nach Örtlichkeit, Lüftungskonzeption und der daraus resultierenden Strömung im Innenraum kann die Lüftungseffizienz von Fall zu Fall unterschiedlich ausfallen.

An Schulen und Bildungseinrichtungen haben RLT-Anlagen Vorteile in Form ihrer einfachen Handhabung, der konsequenteren Umsetzung von Lüftung, eines ganzjährig hohen thermischen Komforts und im Sparen von Heizenergie [31]. Im Rahmen der aktuellen Pandemie haben zusätzlich auch relativ einfach zu konstruierende Abluftsysteme Aufmerksamkeit erreicht [32].

Eine Grundregel aus der Innenraumlufthygiene fordert für Unterrichtsbedingungen eine Zufuhr von Außenluft (Frischluft) in der Größenordnung von 30 m^3^ pro Person und Stunde (z. B. [33]). In stark belegten Klassenräumen mit einem typischen Volumen um 200 m^3^ erfordert dies eine Luftwechselzahl der Größenordnung 3–4,5 h^−1^. (Die Luftwechselzahl gibt an, wie häufig das Luftvolumen im Raum pro Stunde durch unkontaminierte Frischluft zu erneuern ist.) Diese Zahlen beruhen auf langjährigen Erfahrungen in der Innenraumhygiene und im Arbeitsschutz und sind auch als Grunderfordernis zur Unterbindung luftgetragener Infektionen aufzufassen, wobei im speziellen Fall einer aerosolübertragenen Virusinfektion wie durch SARS-CoV‑2 eine höhere Luftwechselzahl empfehlenswert sein könnte; letztlich bleibt es aber aufgrund neuerer Virusvarianten und einer sich entwickelnden Gesamtsituation (auch was die Fortführung der anderen Hygienemaßnahmen wie Abstand und Masken angeht) schwierig, hier konkrete Angaben zu machen oder aussagekräftige Studien durchzuführen, welche die Frage des ausreichenden Luftwechsels abschließend behandeln. Die Arbeitsschutzregel (ASR) A3.6 fordert einen Luftwechsel, bei der eine Kohlendioxidkonzentration von 1000 ppm „in der Zeit der Epidemie wenn möglich unterschritten werden“ sollte [34]. In Räumlichkeiten des Gesundheitswesens sind die Anforderungen wesentlich höher, in Form von Luftwechselraten von 5–8 h^−1^ in Krankenhäusern bzw. einer zusätzlichen Luftzufuhr von 100 m^3^ pro Person pro Stunde in Patientenzimmern [35]. Der zur Vermeidung einer Infektion über den Aerosolpfad notwendige Luftwechsel hängt von der Infektionsdosis des Erregers ab und wird insbesondere bei infektiöseren Virusvarianten höher ausfallen als bei weniger infektiösen. Da momentan noch keine ausreichenden Studiendaten verfügbar sind, um einen „sicheren“ Mindestluftwechsel z. B. für die Deltavariante von SARS-CoV‑2 zu definieren, kann es im Sinne des präventiven Infektionsschutzes geboten sein, höhere Luftwechselraten als nach DIN EN 16798 [33] einzustellen.

Tab. 2 stellt die verfügbaren Möglichkeiten der Raum- und Gebäudelüftung dar. Die Bandbreite technischer Möglichkeiten reicht von zentralen und dezentralen Lüftungsanlagen über ventilatorgestützte Abluftanlagen bis zur in der Regel unmittelbar verfügbaren Fensterlüftung.

**Table 2:** 

Generelle Zielwerte für Frischluftzufuhr: ≥ 30 m^3^ pro Person/Stunde Luftwechselzahl in stark belegten Räumen ≥ 3–4,5 h^−1^ (z. B. DIN EN 16798‑3 [33])			
Maßnahme	Wirkung	Zu beachten	Quellen
Raumlufttechnische (RLT-)Anlage	Stellt kontinuierlichen Luftwechsel im Raum sicher	Zentrale Anlagen versorgen Gebäude (Luftzuführung meist vom Dach)	[31, 36]
	Flächenhafte Zu- und Abführung vermeidet Zugerscheinungen	Dezentrale Anlagen versorgen Einzelräume (Luftzuführung durch Fassade)	
	Quelllüftung^a^ ist tendenziell wirksamer als Mischlüftung^b^	Nachrüstung von dezentralen Anlagen ist einfacher	
	Wärme- und Feuchterückgewinnung gewährleisten hohen Komfort	RLT-Anlagen in Verbindung mit Fensterlüftung werden auch als Hybridlüftung bezeichnet	
Fensterlüftung mit weit zu öffnenden, großen Fenstern	Tauscht Raumluft gegen virenarme Außenluft aus	Temperatur und relative Luftfeuchte sinken; bei Stoßlüftung, aufgrund der Wärmespeicherung des Gebäudes allerdings nur geringfügig	[30, 32, 37–39]
	Das Lüftungskonzept des Umweltbundesamtes (20 min Unterricht, 5 min Stoßlüftung [30]) gewährleistet in der Regel einen deutlichen Luftaustausch	Erfolgen Stoßlüftungen sehr häufig, können Räume auskühlen	
	Lüftung beseitigt unerwünschte Gase (Kohlendioxid, Wasserdampf, flüchtige organische Verbindungen)	Platzbedarf durch weit zu öffnende Fenster	
		Integration der Stoßlüftungen in den Unterricht notwendig	
		Mögliches Behaglichkeitsproblem im Winter	
		Mögliche Belastung durch Lärmquellen im Außenbereich	
Kipplüftung durch Fenster oder Oberlichter	Variante der Fensterlüftung mit reduzierter Öffnungsfläche nach außen	Bei Dauerlüftung geht im Winter viel Wärmeenergie verloren	[32, 39, 40]
	Luftwechsel reicht möglicherweise nicht aus	Mögliche Belastung durch Lärmquellen im Außenbereich	
Ventilatorgestütztes Abluftsystem	Einfaches System auf Grundlage von Quelllüftung	Kann baulich meist schneller realisiert werden als eine RLT-Anlage	[32]
	Wirksamer als einseitige Fensterlüftung	Technische Sicherheit bzw. Lärmbelastung beachten	

^a^Bei Quelllüftung wird kühlere Luft in Bodennähe eingeleitet, durch die im Raum befindliche Wärmequellen (darunter die anwesenden Personen) erwärmt und in Deckennähe ausgeleitet. Quelllüftung ermöglicht eine klar ausgerichtete Luftströmung mit tendenziell geringen Verwirbelungen. Die Lüftungseffizienz ist hierbei potenziell größer als bei der Mischlüftung

^b^Bei der Mischlüftung liegen Lufteinlass und -auslass meist auf einer Seite des Raumes (Wand bzw. Decke). Der Luftaustausch findet somit unter stärkeren Gegenströmungen statt

Folgende Aspekte sind zu beachten:

- *Zeitliche Realisierbarkeit.* Die Gesellschaft fordert in der gegenwärtigen Pandemie rasche Umsetzung bzw. Anwendbarkeit technischer Lösungen. Eine wirksame Fensterlüftung ist in den meisten Schulräumen unmittelbar umsetzbar. Ventilatorgestützte Abluftanlagen lassen sich im Verlauf von Monaten realisieren, wogegen dem Einsatz neuer RLT-Anlagen in der Regel lange Planungsprozesse vorangehen.
- *Wirksamkeit. *RLT-Anlagen bzw. geregelte Lüftungsanlagen sorgen für einen kontinuierlichen Luftwechsel, wogegen die Wirksamkeit von Fensterlüftung (stoßweise, aber auch Dauerlüftung) im Einzelfall äußeren Bedingungen wie der Gebäudegeometrie oder der Temperaturdifferenz zwischen Innen und Außen unterliegt.
- *Komfortaspekte.* Die freie Lüftung durch Fenster wird bei kalten Außentemperaturen oft als unangenehm empfunden. Lüftungsanlagen sorgen hingegen für gleichmäßigere Luftströme und Erwärmung der Außenluft. Fensterlüftung erfordert ein regelmäßiges Öffnen und Schließen der Fenster im Klassenraum, während Lüftungsanlagen ohne großen Nutzeraufwand im Hintergrund laufen. Bei Fensterlüftung besteht auch die Möglichkeit einer Belästigung durch Lärmquellen außerhalb des Gebäudes (z. B. Straße).
- *Kostenaspekte*. Virenarme Außenluft durch Fensterlüftung ist in der Regel ohne zusätzliche Investitionskosten sofort verfügbar; in der kühlen Jahreszeit wird vermehrte Fensterlüftung zu erhöhten Heizkosten führen. RLT- bzw. Lüftungsanlagen erfordern – sofern nicht vorhanden – Planungsvorläufe, mehr oder weniger kostspielige Einbauten sowie eine Einrichtung und Wartung durch fachkundiges Personal.

Durch verstärkte Lüftung können vermehrt Inhaltsstoffe aus der Außenluft ins Gebäude getragen werden. Bei starker Belastung der Außenluft, z. B. in der Nähe von stark befahrenen Straßen oder Staubquellen, kann daher beim Einsatz von RLT-Anlagen eine vorherige Filtration der Außenluft notwendig erscheinen.

### Luftreinigungstechniken

Luftreinigung umfasst eine Reihe von Verfahren und Techniken, die auch im Infektionsschutz zum Einsatz kommen können. Bei Luftreinigungstechniken kann generell zwischen fest installierten Anlagen, als Bestandteil von RLT-Anlagen, sowie mobilen Einzelgeräten unterschieden werden (Tab. 3). Bei fest installierten Anlagen kann entweder verbrauchte Raumluft aufbereitet werden (Umluftprinzip), aber auch Außenluft, wenn deren Bestandteile im Innenraum erwünscht sind.

**Table 3:** 

Maßnahme	Wirkung	Zu beachten	Quellen
Filtration in raumlufttechnischen (RLT-)Anlagen	Filtration durch Gewebefilter bei Anlagen im Umluftbetrieb	Sachgerechter Betrieb und Wartungsintervalle müssen beachtet werden	[42]
	Inaktivierung von Viren durch UV-C-Lampen in Umluftkanälen		
Mobile Luftreinigungsgeräte auf Basis von Partikelfiltern (Filtergeräte)	Entfernen Partikel aus der Mischluft im Raum	Geräuschentwicklung beachten	[30, 41, 43–45]
	Senken die mittlere Konzentration aller Partikel	Kein Ersatz für Lüften Sachgerechte Positionierung erforderlich Wartungsintervalle inkl. Filterwechsel beachten	
	Ausreichende Förderleistung an gereinigter Luft notwendig: ≥ 4-faches Raumvolumen pro Stunde [41]		
Mobile Luftreinigungsgeräte auf Basis von Inaktivierung der Viren (UV‑C, Ionisation, Plasma etc.)	Inaktivierung von Viren in der Mischluft im Raum	Funktionsnachweis für mikrobielle Organismen und unter Realraumbedingungen notwendig	[46, 47]
	Senken die mittlere Konzentration infektionsfähiger Partikel	Im Einzelfall Emission unerwünschter Nebenprodukte beachten	
		Kein Ersatz für Lüften	

Der Verein Deutscher Ingenieure e. V. (VDI) hat im Juli 2021 eine Expertenempfehlung veröffentlicht [41], welche die Funktionsweisen mobiler Luftreiniger wie auch ihre sachgerechte Aufstellung vor Ort thematisiert. Das Papier formuliert auch Anforderungen an die Leistungsfähigkeit mobiler Luftreiniger bezüglich ihrer Wirksamkeit sowie möglicher Begleiterscheinungen. Konkrete Anforderungen sind:

- ausreichende Förderleistung an gereinigter bzw. gefilterter Luft; bei allen Geräten geht man von einer Mindestförderleistung in Höhe des 4‑fachen Raumvolumens pro Stunde aus;
- elektrische und optische Sicherheit (Letzteres im Fall von Geräten mit UV-C-Strahlung);
- bei Filtergeräten: Angaben über die Filterklasse;
- bei UV-C-, Ionisations- und Plasmageräten ist ein Wirkungsnachweise für Mikroorganismen im Realraummaßstab notwendig;
- mobile Geräte sollen so aufgestellt werden, dass die Luft im Raum möglichst frei zirkulieren kann und die Reinigungsleistung der Geräte alle belegten Bereiche des Raumes erfasst;
- möglichst geringe Geräuschentwicklung;
- möglichst geringe Emission unerwünschter Nebenprodukte, wie z. B. Ozon.

Es ist zu beachten, dass die meisten mobilen Luftreiniger die Raumluft lediglich umwälzen und bestimmte Inhaltsstoffe entfernen. Solche Geräte ersetzen nicht die notwendige Zufuhr von wenig kontaminierter Außenluft und entfernen insbesondere nicht das ausgeatmete Kohlendioxid. Deshalb sollte auch beim Einsatz von Luftreinigern jede weitere Lüftungsmöglichkeit genutzt werden. In Räumen, in denen überhaupt keine Lüftungsmöglichkeit über Fenster vorhanden ist und auch keine Lüftungsanlage mit Zufuhr von Außenluft zum Einsatz kommt, sollte grundsätzlich kein Unterricht stattfinden – dies gilt auch unabhängig von der Pandemie.

### Hilfestellung durch Kohlendioxidmessgeräte in Schulräumen

Jeder Mensch scheidet mit der Atmung potenziell virushaltige Partikel aus. Über die Atmung wird gleichzeitig auch Kohlendioxid (CO_2_) exhaliert, sodass der Ansatz naheliegt, die Raumluftqualität mittels einer CO_2_-Messung zu bewerten [48]. Sogenannte CO_2_-Ampeln sind meist recht einfache Messgeräte, mit denen der Lüftungszustand in einem Innenraum bewertet werden kann. Nach der Ad-hoc-AG des Umweltbundesamtes [49] gilt die Raumluftqualität bis 1000 ppm (engl.: „parts per million“, dt.: Anteile pro Million) als hygienisch unbedenklich (grüner Ampelbereich), zwischen 1000 ppm und 2000 ppm als hygienisch auffällig (gelber Ampelbereich) und oberhalb von 2000 ppm als hygienisch inakzeptabel (roter Ampelbereich). Auch die Arbeitsschutzregel A3.6 orientiert sich an der Einhaltung einer CO_2_-Konzentration von 1000 ppm „in der Zeit der Epidemie“ [34]. Unabhängig von mikrobiellen Erregern führen hohe CO_2_-Werte bei den Anwesenden zu bekannten Beeinträchtigungen der Konzentration und Ermüdungserscheinungen [50]. Es muss betont werden, dass man die CO_2_-Konzentration nicht als Maß für das Infektionsrisiko deuten kann. Im Rahmen der COVID-19-Pandemie haben sich CO_2_-Ampeln jedoch als nützlich erwiesen, zielgerichtet zu lüften, bzw. dazu beigetragen, Lüftungsprobleme in bestimmten Räumlichkeiten sichtbar zu machen.

## Abschätzung der Wirksamkeit von Maßnahmen mit Modellen

Im Verlauf der COVID-19-Pandemie hat sich die Ansicht durchgesetzt, dass das Infektionsrisiko nur dann beherrscht werden kann, wenn eine Kombination verschiedener Maßnahmen umgesetzt wird, bei denen Lücken oder Schwächen bestimmter Einzelmaßnahmen durch andere Maßnahmen ausgeglichen werden („Schweizer-Käse-Modell“ [51, 52]). Verantwortliche stehen dabei häufig vor der Herausforderung, entscheiden zu müssen, welche infektionsmindernden Maßnahmen in einer konkreten Situation angemessen und wirksam einzusetzen sind. Hieraus ist ein Bedarf nach leicht anwendbaren Prognosemodellen entstanden, welche die Wahrscheinlichkeit einer Übertragung des SARS-CoV‑2 durch Aerosole in konkreten Szenarien abschätzen.

Mathematische Modelle zur Simulation der Ausbreitung von Viruspartikeln in Innenräumen und einer anschließenden Infektion sind seit 2020 für SARS-CoV‑2 entwickelt worden bzw. werden laufend weiterentwickelt [53–58]. Viele der Modelle sind inzwischen online nutzbar. Den Modellen ist gemein, dass sie die für eine luftübertragene Infektion wichtigen Prozesse beschreiben, wofür in der Regel auch immer starke Vereinfachungen vorgenommen bzw. Annahmen getroffen werden müssen. Zu den Prozessen und Annahmen gehören:

- das Ausatmen virushaltiger Partikel durch eine oder mehrere infektiöse Personen beim ruhigen Atmen, Sprechen, Singen oder bei körperlichen Aktivitäten (Sport);
- die Ausbreitung bzw. Verteilung dieser virushaltigen Partikel im begrenzten Raumvolumen;
- eventuelle Minderungen der Raumluftkonzentration infektiöser Partikel durch Lüftung, Lüftungstechnik, Luftreinigung (fest installiert bzw. mobil), aber auch natürliche Deposition (Absetzen auf Oberflächen) bzw. die allmähliche natürliche Inaktivierung der Viren;
- die Bestimmung der über die Atmung aufgenommenen Menge an Viren bei nicht infizierten Personen;
- die Bestimmung der Wahrscheinlichkeit, dass Personen durch diese aufgenommene Menge an Viren infiziert werden und somit zu Überträgern werden können.

Bei der konkreten Anwendung der Modelle sind zentrale Eingangsparameter durch Nutzerinnen und Nutzer wählbar bzw. werden im Modellansatz vorgeschlagen. Dies betrifft die geplante Anzahl der Personen, ihre Aktivität, Aufenthaltsdauer im Raum, das Raumvolumen und die Art und Qualität der Lüftung, in der Regel ausgedrückt durch die Luftwechselrate. Das Tragen von Mund-Nasen-Bedeckungen (MNB), medizinischen Masken oder Schutzmasken nach Arbeitsschutzstandards (FFP2) wird in den meisten Modellen durch einen Faktor berücksichtigt. Die Berechnung einer absoluten Infektionswahrscheinlichkeit für ein gewähltes Szenario (Schulstunde, Besprechung, Sportunterricht etc.) erfordert eine Annahme für die nur mit Unsicherheiten bekannte Infektionsdosis (engl.: „quanta“). Das Modell der RWTH Aachen [59] beschränkt sich darauf, eine relative Infektionswahrscheinlichkeit im Vergleich zu einem Referenzszenario anzugeben, bei dem Infektionen als sehr unwahrscheinlich angenommen werden. Dies ist im konkreten Fall eine Raumbelegung mit 25 Personen und einer sprechenden Person sowie ein Raumvolumen von 200 m^3^, eine Aufenthaltsdauer von 1 h und einer maschinellen Luftwechselzahl von 4,4 h^−1^ [55]. Eines der Modelle berücksichtigt die Verfügbarkeit einer CO_2_-Messung im Innenraum bei der Risikobewertung [58].

Anwenderinnen und Anwender müssen sich klar machen, dass alle veröffentlichten Modelle weitreichende und teilweise stark vereinfachende Annahmen bezüglich der ablaufenden Prozesse treffen müssen und die absolute Wahrscheinlichkeit, sich in einem bestimmten Szenario mit SARS-CoV‑2 über Aerosole zu infizieren, nur mit beträchtlichen Unsicherheiten zu prognostizieren ist. Starke Unsicherheiten bestehen bezüglich der Annahme zur Infektionsdosis, welche bei Varianten des Virus variieren wird, aber z. B. auch zur (instantanen) Ausbreitung der Partikel im Raum.

Modellergebnisse können Aspekte zur Risikoabwägung beitragen, wenn beispielsweise Prioritäten bei der Nutzung von Räumlichkeiten gesetzt werden müssen, eignen sich aber nicht als alleinige Begründung für den vermeintlich sicheren Aufenthalt in Räumen. Für die Dauer der COVID-19-Pandemie sollten Zusammenkünfte von Menschen in Innenräumen daher auch weiterhin an erster Stelle auf ihre Notwendigkeit geprüft werden.

## Diskussion und Ausblick

Zum Zeitpunkt der Erstellung dieses Artikels gelten laut der US-amerikanischen Centers for Disease Control and Prevention (CDC; [26]) trotz wissenschaftlicher Wissenslücken 3 wesentliche Grundannahmen der Übertragung des SARS-CoV‑2 als gesichert:

1. SARS-CoV‑2 wird durch die Exposition gegenüber respiratorischen Flüssigkeiten übertragen. Diese können in Form unterschiedlich großer Tröpfchen bzw. Aerosolpartikel verbreitet werden und zur Infektion führen.
2. Das Risiko einer SARS-CoV-2-Infektion variiert in Abhängigkeit der Anzahl von Viruspartikeln, denen eine Person ausgesetzt ist. Das Infektionsrisiko nimmt mit zunehmender Entfernung von der Quelle und zunehmender Zeit nach dem Ausatmen ab. Daher sind die Präventionsmaßnahmen der zeitlichen Begrenzung des Aufenthalts in Schulräumen sowie die Einhaltung von Mindestabständen (> 1,5 m) sehr effizient.
3. Die Übertragung von SARS-CoV‑2 durch eine Inhalation des Virus über die Luft kann auch dann erfolgen, wenn sich die Quelle räumlich oder zeitlich nicht im direkten Umfeld der exponierten Person befindet, da sich infektiöse Aerosole in Innenräumen anreichern und längere Zeit verbleiben können, wenn im Raum kein genügender Luftaustausch stattfindet.

Wir erkennen infolge der SARS-CoV-2-Pandemie zwei Herausforderungen bezüglich des Infektionsschutzes mit unterschiedlichen Zeithorizonten: Zum einen geht es darum, an Schulen rasch und kurzfristig eine Situation zu erzeugen, mit der das Infektionsrisiko durch die Aerosolübertragung deutlich reduziert wird, sodass Ausbrüche von SARS-CoV‑2 an Schulen vermieden werden. Dies ist ein dringliches Problem, da Kinder und Jugendliche zum jetzigen Stand (Oktober 2021) oft noch nicht geimpft sind und somit eine ungeschützte Population darstellen. Hierfür kommen kurzfristig realisierbare Maßnahmen infrage wie eine Erhöhung des Luftwechsels bei existierenden RLT-Anlagen, eine Intensivierung der Fensterlüftung in den Klassenräumen, der Einbau von Zu- und Abluftanlagen. Mobile Luftreinigungsgeräte stellen in Zeiten der Krise eine Möglichkeit dar, einen zusätzlichen Schutzeffekt zu erzielen.

Zum zweiten hat die Pandemie die seit Längerem bekannten, aber nur ungenügend wahrgenommenen Defizite bei der Lüftung von Schulräumen in Deutschland [31] offengelegt. Diese strukturellen Schwächen können nur langfristig durch bauliche Maßnahmen behoben werden. Dort, wo über die freie Lüftung (Fenster) kein ausreichender Luftaustausch stattfinden kann, ist die Installation von RLT- oder Lüftungsanlagen sinnvoll, bestenfalls mit Wärme- und Feuchterückgewinnung. Diese Anlagen werden bezüglich ihrer Planung und Umsetzung Zeit erfordern, lassen jedoch neben der förderlichen Verbesserung der Innenraumluftqualität im Allgemeinen auch einen Nutzen beim saisonalen Infektionsgeschehen oder bei künftigen Pandemien erwarten. Im Vergleich zur Fensterlüftung vermeiden RLT-Anlagen Behaglichkeits- und Umsetzungsprobleme bei extrem niedrigen oder hohen Außentemperaturen. Die Wärmerückgewinnung trägt zur Kostenreduzierung der Gebäudeheizung bei.

Ungeachtet der offenen Fragen zu Infektiosität und Übertragungsmodalitäten von SARS-CoV‑2 wird der Lüftung in Innenräumen auch zukünftig eine zentrale Bedeutung zukommen [60]. Dies gilt auch im Angesicht möglicher zukünftiger Virusvarianten, welche ein deutlich höheres Übertragungsrisiko aufweisen können. Der Austausch von Raumluft gegen Außenluft wird in Innenräumen, die von vielen Menschen gleichzeitig benutzt werden, auch zukünftig eine zentrale Rolle spielen, da es gilt, neben infektiösen Viren auch verbrauchte Luft (CO_2_) aus Räumen abzutransportieren.

Infektionen der Atemwege sind generell im Winter häufig und äußern sich üblicherweise in Form einer Erkältungskrankheit. Sehr häufig sind Rhinoviren für solche meist harmlosen Erkrankungen verantwortlich, aber auch viele andere Viren (und auch Bakterien und Schimmelpilzsporen) können in schlecht gelüfteten Räumen übertragen werden bzw. infektiös wirken [61]. Trockene, kalte Luft begünstigt die Stabilität und damit auch die Infektiosität vieler Viren und aller Wahrscheinlichkeit nach auch des SARS-CoV‑2 [62]. Insbesondere Innenraumluft mit niedriger Luftfeuchtigkeit kann im Hinblick auf SARS-CoV-2-Übertragungen problematisch sein [63]. Gleichzeitig führt im Winter die Auskühlung der oberen Atemwege zu einer höheren Empfänglichkeit für Infektionen, weil die Kälteeinwirkung, besonders in Kombination mit einer Austrocknung der Schleimhäute, zu einer reduzierten Abwehr infektiöser Partikel führt [62, 64]. Dennoch kommt die Studie von Aganovic et al. (2021) zu dem Schluss, dass Befeuchtungsmaßnahmen im Innenraum (von 40 % nach 60 %) keinen bedeutsamen Effekt auf die Infektionswahrscheinlichkeit ausüben [65], sondern dass der Lüftung eine Schlüsselrolle zur Reduzierung der Viruskonzentration zukommt. Weltweite epidemiologische Beobachtungen legen zwar mehr Ausbrüche bei feuchtwarmen Klimaten nahe [66], doch bezieht sich dies auf relative Feuchten oberhalb 80 %, die im Innenraum kaum auftreten.

Auch nach Überwindung der SARS-CoV-2-Pandemie können die erlernten Maßnahmen für die Innenraumluftqualität in Schulen dazu beitragen, andere aerosolübertragene Viruserkrankungen zu vermindern. Das Ausbleiben einer Grippewelle im Winter 2020/2021 deutet bereits darauf hin, dass die Präventionsmaßnahmen auch bei anderen Infektionskrankheiten eine Wirkung erzielen: Trotz COVID-19 lag die Prävalenz akuter Erkrankungen der Atemwege während des harten Lockdowns in Deutschland (Ende 2020 bis Ende Februar 2021) auf einem bislang nie dagewesenen niedrigen Niveau [67]. Auch international wurde über eine ungewöhnlich niedrige Influenzaaktivität berichtet, die deutlich unter den Ergebnissen der Vorjahre liegt [68].

Dieser Artikel reflektiert den Stand des Wissens und die Lage der Pandemie im Oktober 2021. Die zukünftige Entwicklung wird auch zeigen, ob aufgrund des Auftretens neuer und evtl. leichter übertragbarer Virusvarianten von SARS-CoV‑2 eine Neubewertung der Lüftungs- und/oder Luftreinigungsmaßnahmen erforderlich sein wird. Dies betrifft des Weiteren auch die Aspekte des Abstands zwischen Menschen in Innenräumen, das Tragen von Masken, allgemeine Hygieneempfehlungen, die Verwendung von Desinfektionsmitteln und letztlich auch die Gestaltung und Nutzungsart von Innenräumen. Möglicherweise könnte SARS-CoV‑2 auch die architektonische Gestaltung und Nutzung von Schulneubauten beeinflussen. In jedem Fall werden zukünftig Fragen der Belüftung, aber auch der Größe und Deckenhöhe von Räumlichkeiten mehr als in den vergangenen Jahrzehnten eine Rolle spielen, denn eine ausreichende Lüftung an Schulen ist ein Schlüssel dazu, die Gesundheit und die Leistungs- und Konzentrationsfähigkeit von Schülerinnen und Schülern bestmöglich zu erhalten und zu fördern.

## Footnotes

1 Der Begriff „Aerosol“ bezeichnet die Mischung von festen oder flüssigen Partikeln in einem Gas oder Gasgemisch (z. B. Luft). Aerosolpartikel treten in der Umwelt in sehr unterschiedlicher Größe und chemischer Zusammensetzung auf und haben Durchmesser von etwa 1 nm bis zu mehreren 100 µm. Aerosole sind generell nicht stabil und können sich im Lauf der Zeit in Abhängigkeit von Luftfeuchtigkeit, Temperatur und weiteren physikalischen und chemischen Prozessen verändern.

2 Zum einen reduziert sich die Wahrscheinlichkeit, dass eine infizierte Person im Raum ist. Zum anderen ist mehr Luftvolumen pro Person verfügbar, in dem sich ausgeatmete Partikel verdünnen können. Darüber hinaus sind größere Abstände möglich, was die Möglichkeit von Infektionen im Nahfeld vermindert.

3 Bei gegebener Konzentration werden über eine kürzere Zeit weniger Aerosolpartikel inhaliert. Zudem ist in einem gelüfteten Raum die Konzentration ausgeatmeter Aerosole zu Veranstaltungsbeginn noch sehr niedrig (im Idealfall null) und nimmt erst mit der Zeit zu.

4 Diese Aktivitäten verstärken die Zahl und Masse ausgeatmeter Partikel um ein Vielfaches [13, 14].

5 Je nach Konstruktion und Gewebetyp halten medizinische (OP-)Masken und dicht anliegende Halbmasken unterschiedliche Anteile ausgeatmeter Partikel und Tröpfchen zurück [29].

6 Je mehr Raumvolumen zur Verfügung steht, umso mehr können sich ausgeatmete Aerosolpartikel ausbreiten und verdünnen, bevor eine stationäre Endkonzentration erreicht wird.

7 Raumlufttechnische (RLT-)Anlagen umfassen zum einen Anlagen, die der reinen Lüftung dienen, aber auch Anlagen, die zusätzlich Einfluss auf die Raumlufttemperatur bzw. -feuchte nehmen [36]. Es wird zwischen „zentralen Anlagen“ für ein Gebäude sowie „dezentralen Anlagen“, die raumscharf eingebaut werden, unterschieden. Lüftungsanlagen können mit Wärmetauschern ausgestattet werden, welche im Winter kalte Außenluft vor Eintritt in den Raum anwärmen und im Sommer warme Außenluft abkühlen.
